# Machine Learning Classifiers to Evaluate Data From Gait Analysis With Depth Cameras in Patients With Parkinson’s Disease

**DOI:** 10.3389/fnhum.2022.826376

**Published:** 2022-05-19

**Authors:** Beatriz Muñoz-Ospina, Daniela Alvarez-Garcia, Hugo Juan Camilo Clavijo-Moran, Jaime Andrés Valderrama-Chaparro, Melisa García-Peña, Carlos Alfonso Herrán, Christian Camilo Urcuqui, Andrés Navarro-Cadavid, Jorge Orozco

**Affiliations:** ^1^Fundación Valle del Lili, Departamento de Neurología, Cali, Colombia; ^2^Fundación Valle del Lili, Departamento de Neurocirugía, Cali, Colombia; ^3^Universidad Icesi, Lab i2t/CENIT, Cali, Colombia; ^4^Fundación Valle del Lili, Centro de investigaciones clínicas, Cali, Colombia; ^5^Universidad Icesi, Facultad de ciencias de la salud, Cali, Colombia

**Keywords:** Parkinson’s disease, gait, biomechanics, kinect, depth camera, machine learning

## Abstract

**Introduction:**

The assessments of the motor symptoms in Parkinson’s disease (PD) are usually limited to clinical rating scales (MDS UPDRS III), and it depends on the clinician’s experience. This study aims to propose a machine learning technique algorithm using the variables from upper and lower limbs, to classify people with PD from healthy people, using data from a portable low-cost device (RGB-D camera). And can be used to support the diagnosis and follow-up of patients in developing countries and remote areas.

**Methods:**

We used Kinect^®^eMotion system to capture the spatiotemporal gait data from 30 patients with PD and 30 healthy age-matched controls in three walking trials. First, a correlation matrix was made using the variables of upper and lower limbs. After this, we applied a backward feature selection model using R and Python to determine the most relevant variables. Three further analyses were done using variables selected from backward feature selection model (Dataset A), movement disorders specialist (Dataset B), and all the variables from the dataset (Dataset C). We ran seven machine learning models for each model. Dataset was divided 80% for algorithm training and 20% for evaluation. Finally, a causal inference model (CIM) using the DoWhy library was performed on Dataset B due to its accuracy and simplicity.

**Results:**

The Random Forest model is the most accurate for all three variable Datasets (Dataset A: 81.8%; Dataset B: 83.6%; Dataset C: 84.5%) followed by the support vector machine. The CIM shows a relation between leg variables and the arms swing asymmetry (ASA) and a proportional relationship between ASA and the diagnosis of PD with a robust estimator (1,537).

**Conclusions:**

Machine learning techniques based on objective measures using portable low-cost devices (Kinect^®^eMotion) are useful and accurate to classify patients with Parkinson’s disease. This method can be used to evaluate patients remotely and help clinicians make decisions regarding follow-up and treatment.

## Introduction

Parkinson’s disease (PD) represents the second most prevalent neurodegenerative disease in the world with an alarming growth rate in the number of affected individuals estimating that the number of cases will double between 2015 and 2040 (de Lau and Breteler, 2006; Tysnes and Storstein, 2017; Dorsey and Bloem, 2018). PD is clinically characterized by motor symptoms such as bradykinesia, rigidity, tremor, gait disturbance, and impaired postural instability (Schneider and Obeso, 2014; Postuma et al., 2015; Deb et al., 2021). Diagnosis and follow-up are based on several scales and questionnaires to assess severity including Movement Disorder Society-Sponsored Revision of the Unified Parkinson’s Disease Rating Scale (MDS-UPDRS; Goetz et al., 2008). However, these clinical scales are subjective with high inter-rater variability between clinicians. Furthermore, follow-up is also based on self-report questionnaires that imply recall bias (Deb et al., 2021). In the last 20 years, there has been great interest in developing objective measurement focused on early diagnosis, accurate follow-up, evaluation of motor fluctuations, and prognosis in PD, from which has arisen technology-based objective measurements (TOMs) as a complement for clinical assessment (Urcuqui et al., 2018; Deb et al., 2021).

In PD, changes in gait kinematics and spatiotemporal features are hallmarks of the disease. Gait analysis is complex and usually requires a gait and biomechanics laboratory which is expensive and not globally available for medical consultation (Urcuqui et al., 2018). Recently, several cost-effective instruments have been used to assess PD motor symptoms such as RGB-D cameras (Kinect^®^). Despite the large number of TOMs studies and available data, such as inertial measurement units (IMUS) that do not need a specialized laboratory, the RGB-D cameras are the most accessible technology in remote areas for its cost and its simplicity. However, the data processing and classification methods are still variable upon the studies.

Machine learning (ML) techniques have been studied in several medical areas including PD (Sidey-Gibbons and Sidey-Gibbons, 2019) in order to classify healthy volunteers from patients using voice analysis (Ozkan, 2016), feet pressure systems (Abdulhay et al., 2018), RGB-D cameras (Buongiorno et al., 2019; Jaggy Castaño-Pino et al., 2019), optoelectronic motion analysis system (Varrecchia et al., 2021), wearable sensors such as accelerometers or inertial measurement units (IMU; Yoneyama et al., 2013; Caramia et al., 2018), walkway pressure analysis (Wahid et al., 2015), and variables associated with knee and trunk rotation (Varrecchia et al., 2021). Other studies have been using unsupervised learning to extract features in the initial stages of the disease (Singh and Samavedham, 2015), propose a method to obtain informative correlation-aware signals (Zhang et al., 2021), and evaluate clustering algorithms to support the prediction of the disease (Sherly Puspha Annabel et al., 2021). Most of the studies that aimed to classify healthy people from PD patients focused solely on leg variables or arm variables or axial trunk and knee rotation even though the disease involves all four limbs and the first affected are the arms (Ospina et al., 2018; Monje et al., 2021).

With the rise of telemedicine in recent years, particularly after the beginning of the SARS-CoV2 pandemic, never has it been so important to develop simple assessment methods that do not require high costs or specialized equipment, particularly in developing countries where access to specialized medicine is limited. In addition, telemedicine programs in Parkinson’s disease are a growing field and gait measurement demands many challenges to evaluate patients in rural regions and developing countries in order to ensure quality evaluation. Remote monitoring with synchronous and asynchronous assessments included the use of specialized devices and recorded and uploaded videos, for motor evaluation such as bradykinesia, gait, and falls (Shalash et al., 2021).

In this work, our aim is to study the causal relationship between gait features from upper and lower extremities and assess the performance of a machine learning model to classify people with PD from healthy subjects using data from a portable low-cost device (Depth Camera) called Kinect^®^eMotion system in order to support diagnosis and follow-up to patients with PD in remote areas.

## Materials and Methods

### Design and Participants

The dataset was extracted from a single-center study carried out between June and December 2016, by the Neurology Service at the Fundación Valle del Lili academic Hospital in Cali—Colombia (Muñoz Ospina et al., 2019). We included spatiotemporal gait data from 30 patients with PD and 30 healthy age-matched controls. Each patient was evaluated by a movement disorder specialist and met the criteria from the UK Parkinson’s Disease Society Brain Bank diagnostic criteria. No participants had major features that affected their gait (major orthopedic surgeries, osteoarthritis, other neuromuscular disorders, or walking aids) All participants with PD were treated with dopaminergic agonists and were evaluated in the “on” state. Institutional review board approval was obtained prior to starting the study and all participants provided written informed consent before participation.

**Figure 1:**
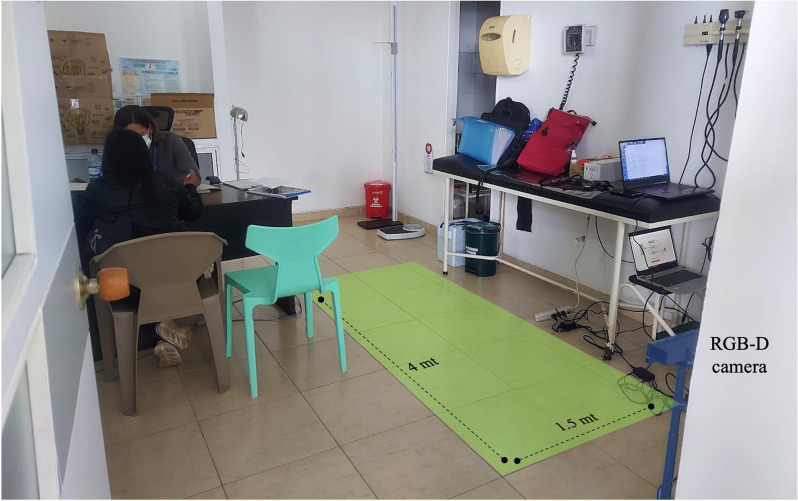
RGB-D camera setup and gait evaluation zone.

Gait data were obtained from previous studies using an RGB-D camera (Kinect^®^eMotion) coupled with a signal processing software. Subjects underwent a single gait evaluation session during which each subject was asked to walk at their preferred speed during three consecutive walking trials. The measurements were made in a corridor 4 m long and 1.5 m wide free of interference. The distance allowed for Kinect^®^ to record a minimum of one full gait cycle per limb. Figure 1 shows the setup during a measurement campaign in a rural area in the southwest of the country.

As indicated in previous studies we used wavelet techniques to extract gait phases and generate several spatio/temporal variables (see Table 1). These variables were obtained based on a wavelet decomposition using a Daubechies wavelet (Db8; Jaggy Castaño-Pino et al., 2019).

**Table 1:** Gait variables definition.

**Arms variables**	
Swing magnitude (left/right)	Distance taken by the wrist from the maximum anterior to the maximum posterior point during an arm swing cycle.
Swing time (left/right)	Time taken by the wrist to travel the distance between the maximum anterior and maximum posterior points during an arm swing cycle.
Swing speed (left/right)	Calculated as the distance traveled by the arm (arm swing) per unit of time (arm time)
Arm swing asymmetry (ASA; Zifchock et al., 2008)	$ASA=\frac{\left[45^{\circ }-arct\left(\frac{Armswingmore}{Armswingless}\right)\right]}{90^{\circ }}\times 100$
**Leg variables**
Global gait speed	Calculated as the distance traveled (test distance) per unit of time (test time)
Total time (left/right)	Time during which the ankle (left/right) was in the motion capture area.
Total distance (left/right)	Distance during which the ankle (left/right) was in the motion capture area.
Total swing time (left/right)	Total time while the foot (left/right) was in the swing phase.
Total stance time (left/right)	Total time while the foot (left/right) was in the stance phase.
Swing time (left/right)	Time, while one foot left/right, was on swing phase during 1 leg gait cycle.
Stance time(left/right)	Time while one foot (left/right) was on stance phase during 1 leg gait cycle.
Number of steps (left/right)	Number of steps taken by one foot (left/right) during the test.
Step length (left/right)	Distance traveled by one foot (left/right) during 1 step.

*ASA: arm swing more is the value of the arm’s swing magnitude with the highest value. It is expressed in percentages*.

### Preprocessing Features

As we aimed to study arms and legs variables (one dataset for each set of features), the integration of all the data was made using a unique ID for each patient and the result was a dataset of 620 records and 28 features. The join presented 96 records without values that were excluded during the study. After the filtering process, the dataset had a shape of 554 × 28; 37% of the dataset corresponded to healthy controls and 63% to PD patients.

Two datasets were generated with the same shape for further analysis: one with normalized information because this is a prerequisite for some machine learning algorithms and the other set of information without normalizing technique. For normalization we used the open library ClusterSim (Walesiak and Dudek, 2020) which uses the following formula:$\frac{\left(x-mean\right)}{\sqrt{(sum{\left(x-mean\right)}^{2}}}$.

### Exploratory Analysis

The data exploration included an evaluation between gait variables using a correlation matrix; three thresholds (0.35, 0.4, and 0.9) were selected randomly from a range of 0–1. Each value was analyzed, the correlations higher than the threshold 0.9 did not show similarity in the variables related to arms and legs. Using the value of 0.4 the features presented a similarity; the highest similarities between upper and lower extremities were obtained using 0.35 as the correlation threshold. Scatter plots were created using the correlation matrix. Backward and forward feature selection models were applied using R and Python to determine the most important variables for further analysis, especially to perform a partial correlation analysis with different sets of features. The significance level selected was 5% for all the variables in the backward feature selection process.

### Machine Learning and Evaluation

Three further analyses were done using variables selected from: Backward models (Dataset A), movement disorders specialist (Dataset B), and all the variables from the dataset (Dataset C).

Dataset A: In order to find the most important variables, a backward elimination process for all the models used in this research was run for the full set of variables and the results were: Left-arm magnitude, arm swing asymmetry (ASA; Zifchock et al., 2008), left swing time, left length of step.

Dataset B: Eight variables were selected (Swing magnitude of both arms, swing time of both legs, step length of both feet, ASA, and global gait speed) by a movement disorder specialist according to their clinical relevance to PD diagnosis and follow-up.

Dataset C: All variables were included in this dataset.

Seven machine learning algorithms were chosen based on the results of previous studies (Urcuqui et al., 2018; Reyes et al., 2019; Alzubaidi et al., 2021). Six of the selected algorithms were trained using R statistical software (logistic regression, decision tree without processing, pre-pruning decision tree, post-pruning decision tree, naive Bayes, and random forest). Using Python, a support vector machine model was trained (see Table 2: machine learning parameters and commands for execution).

**Table 2:** Machine learning parameters and commands for execution.

**Model**	**Dataset C**	**Dataset B**	**Dataset A**
Logistic regression	method=“glmStepAIC” Direction= “forward” preprocess = c (“center”, “scale”) trace = TRUE, AIC = 607.49	method=“glmStepAIC” Direction= “forward” preprocess = c (“center”, “scale”) trace = TRUE, AIC = 621.4	method=“glmStepAIC” Direction= “forward” preprocess = c (“center”, “scale”) trace = TRUE, AIC = 620.67
Support Vector Machine	Kernel = rbf C = 5 Gamma = 0.05	Kernel = rbf C = 10 Gamma = 0.05	Kernel = rbf C = 10 Gamma = 0.05
Decision tree without processing	CP = 0.042	CP = 0.036	CP = 0.027
Decision tree pre-pruning	Depth = 5	Depth = 8	Depth = 7
Decision tree post-pruning	CP = 0.042	CP = 0.036	CP = 0.054
Naïve Bayes	Laplace = 0 UserKernel = TRUE Adjust = 1	Laplace = 0 UserKernel = TRUE Adjust = 1	Laplace = 0 UserKernel = TRUE Adjust = 1
Random Forest	Mtry = 3 nTree = 1500	Mtry = 2 nTree = 1500	Mtry = 2 nTree = 1500

The experiments applied hold-out (a train set, validation set, and testing set were made) and K-fold cross-validation to reduce overfitting. The dataset was divided into: 10 records for final validation, 80% for algorithm training, and 20% for testing. The cross-validation used k iterations equal to 5 to include different sets of information during the training and validation phases. Classification metrics used in this study for the testing phase were accuracy, false-positive ratio, false negative ratio, and Cohen’s Kappa, the latter as an evaluation metric to evaluate the model’s performance against the imbalance of the values from the dependent variables.

### Causal Inference Model

We decided to find if there was some causal relationship between the variables. For this task, we used the DoWhy library (Sharma and Kiciman, 2020) and applied the causal inference model (CIM). The causal model was applied to each relevant variable of the selected dataset.

The DoWhy library is a Python library developed by Microsoft with the aim to spark causal thinking and analysis. The main idea of the DoWhy library is to model and validate causal assumptions testing these assumptions for any estimation method. The library is based on the Structural Causal Model theory proposed by Pearl (1995) and implements a refutation API to simplify the analysis for non-experts in this area (see \hyperref[s10]**Supplementary Material** for details on the procedure).

## Results

We included data from 30 patients with PD, 17 (57%) men, and 30 healthy age-matched controls. Both groups had a median age of 66 years (IQR 59–75). The median duration of the disease was 5 years (IQR 1–7). Hoehn and Yahr stage classification was stage I for 17% of the patients, stage II for 73%, and stage III for the remaining 10%. The mean of MDS-UPDRS part III was 39.06 (±13.74; see Table 3). We retained a dataset with 554 records and 28 variables, we did not exclude outliers to simulate real clinical situations.

**Table 3:** Clinical features of the sample.

	PD patients	Healthy controls	*p*-value
Age	66 (IQR 59–75)	66 (IQR 59–75)	0.88
Sex:	17 (57%)	19 (63%)	0.60
Male	13 (43%)	11 (36%)	
Female			
Disease duration (years)	5 (IQR 1–7)	-	
Hoehn and Yahr		-	
I	5 (17%)		
II	22 (73%)	-	
III	3 (10%)	-	
MoCA	22 (IQR 16–26)	22.5 (IQR 21–24)	0.57
Left side symptoms	17/30 (57%)		
Right side symptoms	11/30 (37%)		
Symmetrical	2/30 (7%)		
MDS-UPDRS III	39.06 (±13.74)	-	
FOG-Q	6.73 (± 4.95)		

*MoCA, Montréal cognitive Assessment; FOG-Q, Freezing of gait questionnaire. MDS-UPDRS part III: movement disorder society sponsored revision of the Unified Parkinson’s Disease Rating Scale. The laterality of the symptoms was obtained based on the MDS-UPDRS part III*.

**Figure 2:**
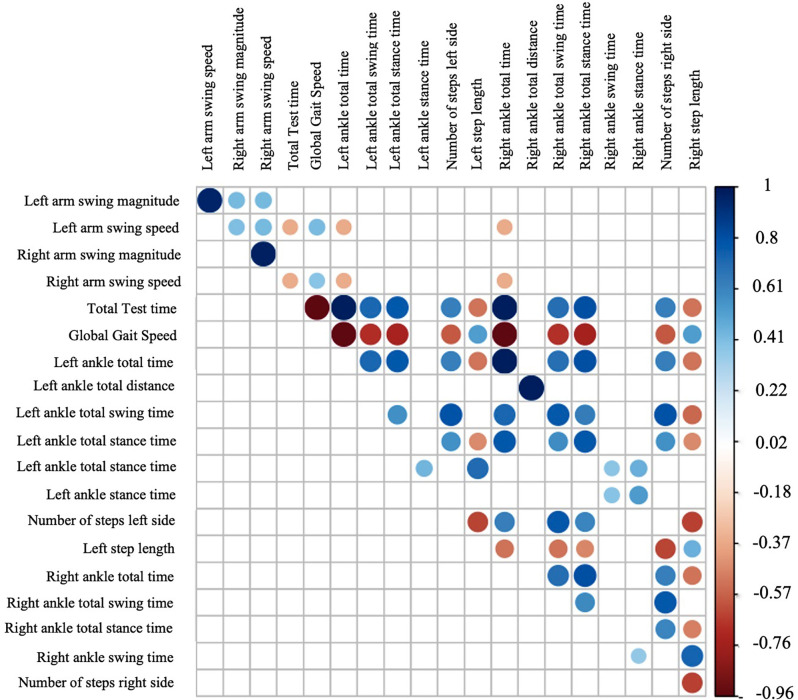
Correlation matrix using gait variables.

First, we conducted an exploratory analysis using a correlation matrix to identify the most relevant variables. We reduced data based on the degree of correlation, retaining only variables with a correlation greater than 0.35 (see Figure 2).

**Figure 3:**
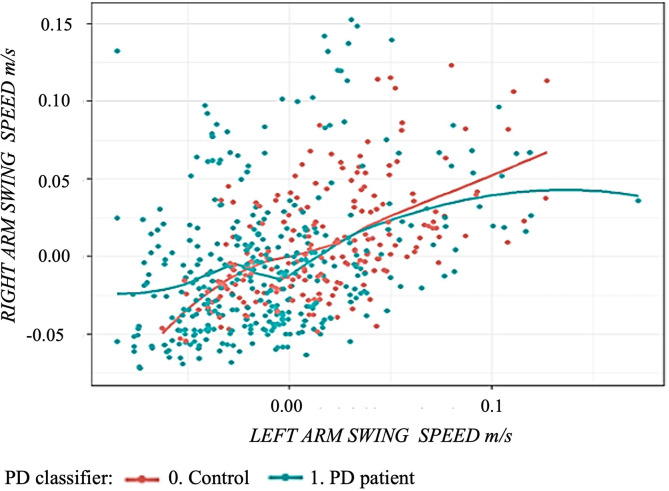
Scatter plot using arm swing speed comparing controls and Parkinson’s disease patients: green curve represents the regression curve of the left and right arms swing speed in Parkinson’s disease patients. The red curve represents the regression curve of the left and right arms swing speed in healthy subjects.

Based on the correlation matrix we obtained several scatter plots (see Figure 3).

### Variable Selection

Using the Backward feature selection model, the most relevant variables were: (1) swing magnitude of left arm; (2) swing time of left leg; (3) left step length; and (4) arm swing asymmetry (ASA). Based on previous studies and clinical expertise, a dataset was created (B) to perform further analysis with some selected variables: swing magnitude of both arms, swing time of both legs, step length of both feet, arm swing asymmetry (ASA), and global gait speed.

### Machine Learning Results

Results from the coefficient of concordance Kappa and accuracy for each model using each set of variables for the test dataset are shown in Table 4.

As we can see, the Random Forest model is the most accurate for all three variable Datasets (Dataset A: 81.8%; Dataset B: 83.6%; Dataset C: 84.5%) followed by the support vector machine for both, A and B datasets, and decision tree pre-pruning for dataset C. Results showing the degree of false positive and false negative are shown for each model and each set of variables in Table 4.

**Table 4:** Confusion matrix results showing kappa, accuracy, false positive, and false negative rate for each machine learning model using the test dataset.

	**Kappa**			**Accuracy**			**False positive**			**False negative**		
	**A**	**B**	**C**	**A**	**B**	**C**	**A**	**B**	**C**	**A**	**B**	**C**
Logistic regression	0.433	0.415	0.400	0.745	0.736	0.718	0.304	0.324	0.381	0.234	0.237	0.221
Support vector machine	0.487	0.572	0.347	0.766	0.802	0.706	0.301	0.204	0.259	0.432	0.323	0.373
Decision tree without processing	0.404	0.388	0.268	0.727	0.718	0.654	0.352	0.369	0.466	0.233	0.237	0.269
Decision tree pre-pruning	0.404	0.471	0.476	0.727	0.745	0.745	0.37	0.362	0.368	0.188	0.175	0.164
Decision tree post-pruning	0.449	0.388	0.268	0.681	0.718	0.654	0.429	0.369	0.466	0.25	0.237	0.269
Naïve Bayes	0.466	0.423	0.414	0.745	0.718	0.690	0.356	0.4	0.448	0.185	0.184	0.094
Random Forest	0.611	0.650	0.661	0.818	0.836	0.845	0.244	0.22	0.167	0.25	0.131	0.149

*A = model using backward selected variables; B = model using expertise selected variables; C = model using all gait variables*.

In order to verify the accuracy of the model we selected 10 aleatory data from the sample (validation records), we compared the classification between patient and control that the algorithm was able to predict vs. the real diagnosis. The accuracy was 90% with only one false positive case.

### Relationship Between Arms and Legs Variables

**Figure 4:**
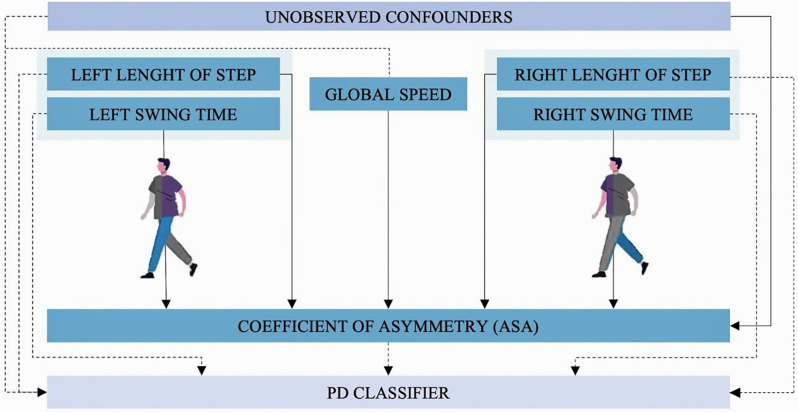
Causal inference model. Dashed arrows show the causal relation identified by the model between leg and arms gait variables with the diagnosis of PD (PD-Classifier). Continued lines show the causal relations identified by the model between arms and legs gait variables.

Due to its accuracy (83.6%) and simplicity (eight variables), dataset B was chosen to run the CIM. Using this model and the DoWhy library relationships between leg gait variables and arm swing variables were analyzed (Figure 4).

Causal inference estimator results show that there is a proportional relationship between ASA and the diagnosis of PD (estimator: 1,536). This can be interpreted as every time the classifier goes up 1 unit (the subject is diagnosed with PD), ASA goes up 1,536 units. To verify the robustness of this estimation three refuters tests were calculated. When “random common cause refuter” is applied to this estimator the results do not significantly vary (1,520), the same happens with “data subset refuter” (1,531) which implies the result is robust. In the placebo treatment refuter, the result for ASA is 0.00436 which is very close to 0. This also means the estimator is robust (see Table 5). That is why based on the DoWhy library, ASA is the most representative variable in the causal inference model.

**Table 5:** Causal inference model estimators and refuters.

	**Estimator**	**Refuter**		
		Random common	Placebo treatment	Data subset
Left arm swing magnitude	−1.403	−1.397	0.011	1.415
Right arm swing magnitude	−1.791	−1.732	0.045	−1.812
Arm swing asymmetry	1.537	1.521	0.005	1.531
Global Gait speed	−0.637	−0.638	−0.021	−0.638
Left foot swing time	−0.109	−0.103	−0.008	−0.110
Left foot step length	−0.464	−0.464	0.010	−0.464
Right foot swing time	−0.120	−0.124	−0.003	−0.111
Right foot step length	−0.386	−0.390	−0.015	−0.389

## Discussion

The main objective of this study was to propose a machine learning-based algorithm to classify the patients with PD from the healthy controls, using a portable RGB-D camera (Kinect^®^eMotion capture system). These results are in line with our attempt to explore other ways to assess the gait variables using a low-cost system that can be used during medical consultation in a developing country. According to our previous results, this machine learning-based algorithm will improve the data analytical and clinical efforts to analyze disease-relevant information for physicians and patients.

### Correlations and Variable Exploration

As expected there is a positive strong correlation between arm speed and arm swing magnitude which represents that some of the normal dynamics of human gait is preserved even in PD patients. Despite the correlation of magnitude between both arms being weak and positive, this could be explained by the limb movement asymmetry secondary to the motor symptoms of the disease (increased rigidity and bradykinesia) predominantly affecting only one body side in the PD group. This pathological asymmetry between left and right arm swing magnitudes is represented by the ASA coefficient which is one of the earliest clinical manifestations of PD (Mirelman et al., 2016).

Regarding the results of the non-PD group, controls exhibit a similar speed in both upper limbs, which could be related to the normal pattern of gait unaffected by the disease (see Figure 3).

### Variable Selection and Dataset Construction

Variables were selected according to different criteria into three datasets. When the backward technique was applied predominantly left variables (arm swing magnitude, step length, and swing time) were selected, which could be related to the prevalence of left-sided motor symptoms in our sample of PD patients (17/30; 57%).

Also, the gait variables selected by the backward feature selection model are related to the clinical changes expected in PD and features needed to fulfill diagnostic criteria: PD patients move their arms and legs more slowly (bradykinesia) and stiffy (rigidity) than controls, for this reason, the magnitude of the arm swing, the time of the leg swing and the step length differ significantly from the healthy-controls.

Furthermore, the selection of both arm and leg variables suggests alterations in the motor pattern of upper and lower limbs. These complex changes in the gait dynamics indicate that objective examination of gait should consider multiple motor variables of each limb. This consideration is consistent with clinical environments where the patient diagnosis and follow-up are based on a full-body examination using the MDS-UPDRS part III (Goetz et al., 2008; Postuma et al., 2015).

### Machine Learning Algorithm

Our results show that it is possible to classify patients from controls using different datasets processed by multiple machine learning techniques with different accuracy levels.

Although dataset C had the best performance, dataset B was chosen for having a high accuracy with a low number of variables, which facilitates the data acquisition and processing.

The clinician accuracy for the diagnosis of Parkinson’s disease varies upon studies, however a systematic review showed that clinical diagnosis for PD in non-experts is 73.8% (67.8%–79.6%); for a movement disorder expert at first consult is 79.6% (46%–95.1%) and 83.9% at follow-up (69.7%–92.6%). Also, the accuracy for the UK Parkinson’s Disease Society Brain Bank diagnostic criteria is 82.7% (62.6%–93%; Rizzo et al., 2016) with a high sensibility (90%) but a low specificity (30%–40%; Marsili et al., 2018). With an accuracy of 83.3%, the selected random forest machine learning algorithm is not far from the clinical reality in the ideal settings. These selected variables are closely related to the PD diagnostic criteria because they represent surrogate measures of the slowness of movement (bradykinesia), asymmetry of arm swing, and rigidity.

### The Gait Is Intricate: The Causal Inference Estimator

Although much is known about the gait pattern, asymmetry of arm swing (ASA) is a clinical characteristic that has been widely used in the last decade to describe the affected motor central pattern in PD patients (Lewek et al., 2010; Huang et al., 2012; Roggendorf et al., 2012; Mirelman et al., 2016). According to our causal inference estimator, there is a relation between leg variables and the symmetry of the arms which represents a new opportunity in the research of these dynamics, particularly in pathological conditions such as PD.

### Finding Differences Between PD Patients and Controls

As seen in the causal inference estimator, there are some unobserved confounders and other variables that could explain some of the changes secondary to PD, as seen in other neurodegenerative diseases, its complexity, and inter-patient variability difficulties to obtain higher accuracy levels. The challenge of new methods of signal processing and machine learning in clinical research is helping clinicians to achieve clinically meaningful technology-based objective measures (TOMs; Espay et al., 2016).

### Related Work

Prior works were made using RBG-D cameras to classify PD patients, the variables selected included stride length, age, gait speed, stance time, step length, distance, cycle time, and swing time. The model that had the best accuracy (82%) was Random Forest. This includes a larger number of variables and not all of them are related to the clinical reality, also no further analysis was made (Urcuqui et al., 2018).

As reported in the literature other studies used RBG-D cameras to classify PD patients, but they used other methods. One of them used neural networks and cross-validation using the variables of gait velocity and stride length with an accuracy of 97.2% (Ťupa et al., 2015) or another classification method (the Bayesian), with a maximum accuracy of 94.1% using the stride length and age (Procházka et al., 2015). Differences could be also due to different preprocessing, filtering and exploration of the data. However, other models reported in the literature used only variables from legs.

Other studies used foot pressure sensors and selected the variables of stride time, stance time, swing time, and foot strike profile to classify the controls from the PD patients with an accuracy of 92.7% (Abdulhay et al., 2018). Similar accuracy (92.6%) was found with a normalized multiple regression and Random forest using stance time, stride length, time of total stance, and cadence with the same type of device (Wahid et al., 2015).

The arm swing analysis has been a point of interest in the study of PD. Previous studies confirm that the arms swing magnitude and speed are significantly reduced in the PD for both limbs (Jaggy Castaño-Pino et al., 2019). On the other hand, several studies have been made with wearable technology (Inertial movement unit (IMU), accelerometers). An arm swing asymmetry (ASA) can also be extracted with accelerometers, it is calculated with the root mean square (RMS) differences between arm movements. The ASA and RMS significantly differ in PD patients. This could be used in future studies (Rincón et al., 2020).

### Advantages, Limitations, and Future Work

The Kinect^®^eMotion system is a portable RGB Camera that can be used in different scenarios (Figure 1) and does not require a specialized gait laboratory. For that reason, this technology can be used as a complement to telemedicine in places without specialized medicine to support the diagnosis and management of patients’ PD. Our findings suggest that in the future it could be considered to employ these measures and algorithms to complement Parkinson’s disease diagnosis as well as to adapt the algorithms to evaluate disease progression, clinical subtypes, follow-up, response to treatment and correlate with clinical rating scales such as MDS-UPDRS.

Some limitations of the study were the sample size which limited the training of the algorithms to create a more accurate and robust model and only one dataset was used for training the algorithms which could also limit the results. Also, no gait speed matching procedure was implemented, however, some spatio-temporal gait parameters are speed-dependent which may have led to overrepresenting some of the gait variables in the backward feature selection model. Furthermore, some machine learning algorithms described in previous studies for classification between PD and healthy controls were not implemented such as artificial neural networks (ANN) and K-nearest neighbor (K-NN). The first was implemented in the first stages of the study, however, their results were similar to simple statistical methods with no machine learning and no further analysis was performed. The latter is a different machine learning algorithm because it does not save information, it cannot be trained. These limitations will be considered in the development of future studies.

Further studies are needed to explore the use of RGB-D cameras and machine learning algorithms for follow-up and treatment response and more data is needed to improve the machine learning training which will allow to achieve higher accuracy.

## Conclusions

This study shows how machine learning techniques based on objective measures using portable low-cost devices (Kinect^®^eMotion) are useful to classify patients with Parkinson’s disease. This proposed method can be used to evaluate patients remotely and help clinicians make decisions regarding follow-up and treatment.

## Data Availability Statement

The data analyzed in this study is subject to the following licenses/restrictions: the raw data supporting the conclusions of this article can be made available by the authors on request, prior approval by the institutional ethics committee. Requests to access these datasets should be directed to beatriz.munoz@fvl.org.co.

## Ethics Statement

The studies involving human participants were reviewed and approved by Comité de ética en investigación biomédica (CEIB), Fundación Valle Del Lili. The patients/participants provided their written informed consent to participate in this study.

## Author Contributions

JO, AN-C, BM-O, and CU participated in the design of the study, interpretation and revision of data. JV-C organized the database. MG-P, CH, and CU contributed with data processing, statistical analysis, machine learning data processing, and interpretation of the work. JV-C, HC-M, and DA-G participated with data analysis and its interpretation as well as the writing of the first draft of the manuscript. AN-C and BM-O wrote sections of the manuscript. All authors contributed to the article and approved the submitted version.

## Conflict of Interest

The authors declare that the research was conducted in the absence of any commercial or financial relationships that could be construed as a potential conflict of interest.

## Publisher’s Note

All claims expressed in this article are solely those of the authors and do not necessarily represent those of their affiliated organizations, or those of the publisher, the editors and the reviewers. Any product that may be evaluated in this article, or claim that may be made by its manufacturer, is not guaranteed or endorsed by the publisher.
