# Supplementary material for: Machine Learning Classifiers to Evaluate Data From Gait Analysis With Depth Cameras in Patients With Parkinson’s Disease
Source: Front Hum Neurosci. 2022 May 19;16:826376. doi: 10.3389/fnhum.2022.826376 (PMC9160309; doi:10.3389/fnhum.2022.826376)
Supplement: Supplementary file 1 [file Data_Sheet_1.docx]

**SUPPLEMENTARY MATERIAL.**

**Brief explanation of DoWhy algorithm, using causal Inference**.

Causal analysis has been in development process since years ago, and is a tool that goes beyond traditional statistical analysis like regression, estimation, and hypothesis testing techniques. Causal analysis goes one step further; its aim is to infer not only beliefs or probabilities under static conditions, but also the dynamics of beliefs under changing conditions, for example, changes induced by treatments or external interventions. The model we used in the paper is based on the python implementation of the doWhy library, which is support on the Structural Causal Model theory proposed by Pearl in 1995 , used for causal analysis in health. (1–3)

The DoWhy library provides an interface for causal inference analysis with some specific characteristics to simplify the process for non-experts. One of the main characteristics of the library is the refutation API, which automatically tests the causal assumptions to refute it. Also, the library includes an estimation of the conditional effect (CATE) using the EconML library.(4)

**Steps for implementation:**

To build a graph that cover all possible paths related to the effect (it is called the backdoor criterion). In our case, we use two different scenarios as shown in figures. Modeling of the causal problem, Identification of an objective estimate, estimation of the causal effect and refutation of the estimate.

**a. Modeling:** Each causal assumption is made explicit by a graph.

**b. Identify:** Through the graph obtained in the modeling, the best expression for the causal estimation is found.

**c. Estimate:** Provides a causal estimate for the expression found in point b.

**d. Refute:** Verify that the estimate is robust or sound. For the refusal phase, we used three refuters, as authors suggests.

**Add random values or random common cause**

After running the model with additional random causes, the estimates do not change significatively, indicating that the cause or selected “treatment” is robust. In our case, the ASA. The code used was: refute_results=model.refute_estimate (identified_estimand, estimate,method_name="random_common_cause".

**Replaced treatment by a placebo test**

The treatment was permuted, as placebo test and then the analysis was rerun. As the causal estimate changes and is close to zero, then we can infer that our “treatment” was adequate. The code used was: res_placebo=model.refute_estimate(identified_estimand, estimate, method_name="placebo_treatment_refuter", placebo_type="permute")

**Data subset refuter**

The third refuter used was the “data subset”, which consists of divide the data in subsets and rerun the analysis. Results again indicate that the selected “treatment” is adequate. The code used for this refuter was: res_subset=model.refute_estimate(identified_estimand, estimate, method_name="data_subset_refuter", subset_fraction=0.9).

All the refuters used was implemented in the library and used according to examples and documentation.

The following causal probabilities were raised:

**• P (Magnitude_left | do (ret_total_velocity, ret_time_left_swing, ret_length_left_step, ret_time_right_swing, ret_length_right_step))**

**• P (Magnitude_right | do (ret_total_velocity, ret_time_left_swing, ret_length_left_step, ret_time_right_swing, ret_length_right_step))**

**• P (asim_coef | do (ret_total_velocity, ret_time_left_swing, ret_length_left_step, ret_time_right_swing, ret_length_right_step))**

**• P (ret_velocity_total | do (Magnitude_left, Magnitude_right, asim_coef))**

**• P (ret_time_left_swing | do (Magnitude_left, Magnitude_right, asim_coef))**

**• P (ret_length_left_step | do (Magnitude_left, Magnitude_right, asim_coef))**

**• P (ret_time_right_swing | do (Magnitude_left, Magnitude_right, asim_coef))**

**• P (ret_length_right_step | do (Magnitude_left, Magnitude_right, asim_coef))**

All the variables by limb were selected to establish the relationship between the upper and lower limbs. Then, the variables were crossed with each variable of the opposite limb to find causality between them. After this procedure, it was possible to obtain the models that has a better result.

To have a better understanding, a causal graph was created (Figure 1A and Figure 2A), in which the following assumptions are presented:

1. The total Speed ​​that the patient takes to walk “ret_velocidad_total” + the Time that the swing phase of the left foot takes “ret_time_left_swing” + the Length of the step of the left leg “ret_length_left_step” + the Time that The right foot swing phase takes “ret_time_right_swing” + the Right Leg Step Length “ret_length_right_step”, would affect the Arm Swing Asymmetry Coefficient “asim_coef” or not, which could also lead to the Parkinson's disease classifier.


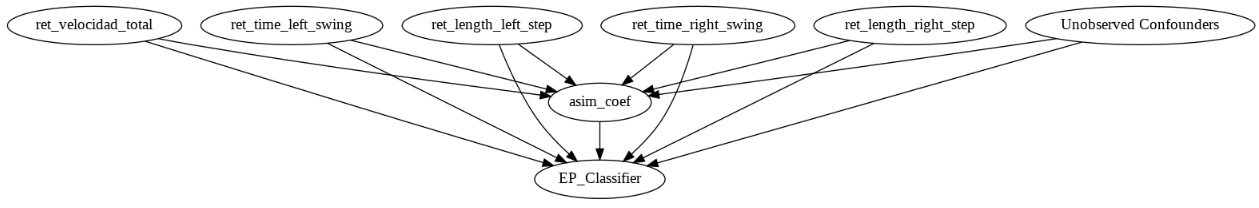


Figure 1A. Graph for asim_coef as "treatment"

1. The anteroposterior displacement of the left arm “magnitud_left” + the anteroposterior displacement of the right arm “magnitud_right” + the asymmetry coefficient of the swing of the arms “asim_coef”, would affect the Step Length of the right leg “ret_length_right_step” or not, which could also lead to Parkinson's Disease classifier.


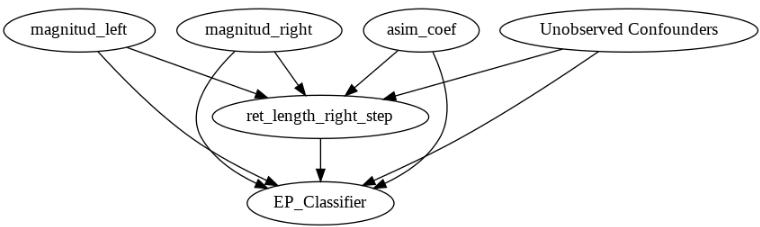


Figure 2A. Graph for the assumption of ret_length_right_step as "treatment"

REFERENCES:

1. Pearl J. Causal diagrams for empirical research. Biometrika. 1995;82(4):669–88.

2. Pearl J. Causality: models, reasoning, and inference. Cambridge, U.K. ; New York: Cambridge University Press; 2000. 384 p.

3. Pearl J. Causal inference in the health sciences: a conceptual introduction. Health Serv Outcomes Res Methodol. 2001;2(3/4):189–220.

4. Sharma A, Kiciman E. DoWhy: An End-to-End Library for Causal Inference. ArXiv201104216 Cs Econ Stat [Internet]. 2020 Nov 9 [cited 2021 Nov 30]; Available from: http://arxiv.org/abs/2011.04216
